# Quantifying microbiota impact on plant traits for the guidance of breeding programs

**DOI:** 10.1111/nph.71108

**Published:** 2026-03-26

**Authors:** Manuel Blouin, Olivier Crépin, Cécile Blanchard, Milena Gonzalo, Olivier Lamotte, Samuel Jacquiod

**Affiliations:** ^1^ Agroécologie Institut Agro, INRAE, Université Bourgogne Europe Dijon France; ^2^ UMR Ampère, CNRS, Ecole Centrale de Lyon, INSA Lyon Université Claude Bernard 16 rue Raphael Dubois 69622 Villeurbanne Cedex France; ^3^ UMR CSGA Institut Agro, CNRS, INRAE, Université Bourgogne Europe 21000 Dijon France

**Keywords:** breeding program, G × E × M, pathogen resistance, plant genotype, plant‐microbiota interactions, shoot biomass, soil environment

## Disclaimer

The New Phytologist Foundation remains neutral with regard to jurisdictional claims in maps and in any institutional affiliations.

## Introduction

Understanding the drivers of plant phenotypic variation is central to crop improvement. Root‐associated microbiota are increasingly recognized as important drivers of plant growth and disease resistance, yet their quantitative contribution to phenotypic variation remains unclear. Here, we extend the classical genotype–environment framework by explicitly incorporating soil microbiota as a distinct factor. By combining contrasting genotypes, soil matrices and microbiota in a full‐factorial experiment, we show that microbiota contribute substantially to plant trait variation, either directly or through interactions with genotype and environment, depending on the trait. We conclude by discussing the implications of these direct and interaction effects for the design of plant breeding programs.

Understanding the sources of phenotypic variation in plants is central to both fundamental biology and agricultural innovation. Classically, plant phenotype (P) is modeled as the additive and interactive effects of plant genotype (G) and the environment (E), formalized as P = G + E + G × E (Falconer, 1989). This framework underlies most quantitative genetic approaches, allowing identification of loci associated with important traits. However, a substantial fraction of organism phenotypic variance often remains unexplained by genetic information alone – a gap referred to as ‘missing heritability’ (Maher, 2008). A convincing explanation could be that, besides plant's genes, additional information sources can be inherited across generations; the so‐called ‘inclusive heritability’ (Danchin *et al*., 2011). In that context, one increasingly acknowledged, yet rarely mobilized, contributor to phenotypic variance is plant‐associated microbiota (Lemanceau *et al*., 2017). Specifically, soil microbial communities are known to influence plant physiology, growth, and health, hence being now proposed by microbial ecologists as a promising way to breed plants (Wei & Jousset, 2017; Trivedi *et al*., 2022). However, plant geneticists are reluctant to incorporate microbiota into breeding programs, given the already extensive experimental designs involved and the limited knowledge of how strongly microbiota influence plant phenotype compared to plant genotype.

Soil microbiota is often considered to be entirely part of the environment (E), due to its strong correlation with soil physicochemical parameters (pH, organic matter, etc.) (Bahram *et al*., 2018; Delgado‐Baquerizo *et al*., 2018). However, rhizosphere and endosphere microbiota were found to be strongly influenced by host genotype, consistently across different soils, giving rise to the concept of a ‘core microbiota’ (Turnbaugh *et al*., 2009; Lundberg *et al*., 2012; Lemanceau *et al*., 2017). Other studies have highlighted that both plant species and soil parameters contribute to shaping the rhizosphere microbiota (Berg & Smalla, 2009), indicating that it cannot be attributed solely to either the environment or the plant genotype. Moreover, microbial inoculations can alter microbial community structure and biomass (Li *et al*., 2024), as well as plant growth (Schütz *et al*., 2018), particularly when consortia rather than single strains are used (Liu *et al*., 2023). These findings indicate that microbiota are influenced by factors beyond soil properties or host genotype, such as agricultural practices and microbial interactions, justifying their consideration as a factor capable of shaping plant phenotype independently of soil properties and host genotype. In this perspective, quantifying the proportion of plant phenotypic variance attributable to microbiota (M), beyond the traditional effects of genotype (G) and environment (E), is a central challenge. The proportion of host trait variance explained by the microbiota has been conceptualized as ‘microbiability’, referring specifically to the additive main effect of microbial communities on host phenotype (Difford *et al*., 2018) and proved successful for quantifying microbiota effects in animal breeding (Camarinha‐Silva *et al*., 2017; Buitenhuis *et al*., 2019). Hereafter, we explicitly distinguish additive microbiability (M) from microbiota‐driven interaction effects (G × M, E × M and G × E × M), which capture complementary, non‐additive pathways by which microbiota modulate host phenotype in a genotype‐ and environment‐dependent manner.

Here, the classical model was enhanced to include the microbiota (M), yielding the extended formula: P = G + E + M + G × E + G × M + E ×  M + G × E × M (Oyserman *et al*., 2021). This captures the idea that microbiota can act as an independent factor (M), but also as a factor dependent on host genotype (G × M), environment (E × M), or both (G × E × M). This distinction has major implications for both plant (Nerva *et al*., 2022; Shen *et al*., 2024) and microbial breeding (Mueller & Linksvayer, 2022). If microbial effects are largely additive (M), they may be harnessed through broad‐spectrum microbial inoculants, efficient across plant genotypes and environments. Conversely, significant G × M or E × M interactions imply that microbial efficacy depends on plant genotypes or environmental conditions, requiring more targeted strategies – like pairing specific plant varieties with tailored microbial consortia. Finally, strong G × E × M interactions considerably hamper broad‐spectrum microbial solutions, requiring the screening of a wide range of inoculants across numerous ‘host genotype × environment’ combinations.

Here, we assessed the microbiability and microbiota‐driven interaction effects of two key traits of *Arabidopsis thaliana* – shoot biomass and pathogen resistance (*Botrytis cinerea*), measured through necrosis size. In a full‐factorial glasshouse experiment, we manipulated the three factors – G (three accessions: Can for Canary Islands; Col for Columbia; Cvi for Cape Verdi Islands), E (three autoclaved soils from Burgundy: Auxonne, Breteniere, Champdôtre) and M (three entire microbial communities extracted from the three soils before autoclaving, used as inoculants) — to evaluate if microbiota's effect on plant was dependent (or not) on the plant genotype and/or the abiotic environment (Supporting Information Fig. S1). At the end of the experiment, we checked that inoculated microbiota changed the structure of the bacterial and fungal rhizosphere communities. We also verified that our design had enough replicates to detect significant effects thanks to a power analysis and quantified the effects of G, E, M and their interactions on shoot dry biomass and leaf necrosis size.

**Fig. 1:**
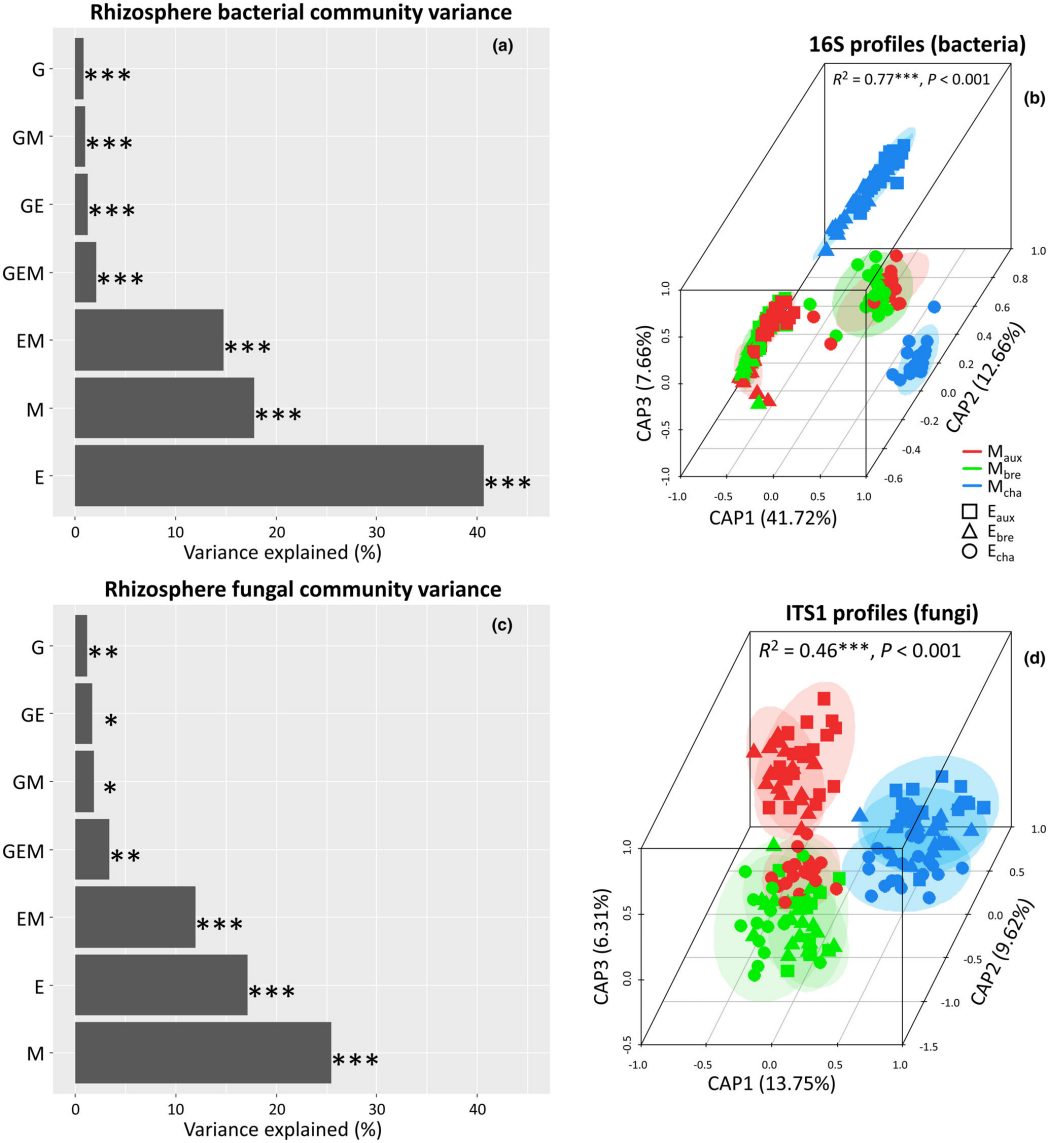
Community structure of bacteria (a, b) and fungi (c, d). Proportion of variance explained by plant genotype (G), soil (E), and inoculated microbiota (M) alone or in interaction (a, c). Distance‐based redundancy analysis (db‐RDA), based on a constrained model including G, E, and M (Bray–Curtis ~G × E × M, 10000 permutations) (b, d). The different colors (red, green, and blue) represent the different microbiota (MAux, Auxonne; MBre, Bretenière; MCha, Champdôtre) inoculated on each genotype and soil. The different shapes (squares, triangles, and circles) represent the three soils. Due to their limited contribution to the model, and for clarity sake, we did not identify the plant genotypes. *n* = 245 (16S‐rRNA) and *n* = 161 (ITS1). Significance code: *, *P* < 0.05; **, *P* < 0.01; ***, *P* < 0.001.

The bacterial and fungal rhizosphere communities at the end of the experiment were primarily shaped by M and E, with G playing a minor but significant role (Fig. 1a,c). For bacteria, E had the strongest influence on explained variance (40.8%, *P* < 0.001), followed by M (17.7%, *P* < 0.001) and the E × M interaction (14.7%, *P* < 0.001), for a total of 73.2% with only these two factors and their interaction (Fig. 1a; Table S1). For fungi, M had the strongest effect (25.6%, *P* < 0.001), followed by E (17.2%, *P* < 0.001) and the E × M interaction (11.8%, *P* < 0.001), for a total of 54.6% with only these two factors and their interaction (Fig. 1c; Table S1). Specifically, for the effect of E, E_Champdôtre_ strongly influenced bacterial communities (Fig. 1b, circles apart from triangles and squares for E_Bretenière_ and E_Auxonne_), and fungal communities to a lesser extent (Fig. 1d). This finding aligns with recent work underlying the major role of soil abiotic environment in reshaping transplanted microbial communities. For instance, Bamba *et al*. (2024) observed that E (e.g. salt‐treated vs control media) significantly shaped root (and not rhizosphere) microbiota variance by 22%. Regarding the effect of M, bacterial communities after inoculation by M_Champdôtre_ were strongly segregating from those inoculated with M_Bretenière_ and M_Auxonne_ (Fig. 1b). Fungal communities that received M_Champdôtre_ or M_Bretenière_ were more stable across environments than those inoculated with M_Auxonne_ (Fig. 1d). This also echoes previous findings (Bamba *et al*., 2024) that M (microbial inoculants from adjacent salt‐treated or untreated fields) was responsible for 22% of root microbiota variance. G effects on rhizosphere microbiota were significant but minor (bacteria: 0.76%, fungi: 1.10%, Fig. 1a,c; Table S1), close to previous findings showing 4% of root microbiota variation due to G (Bamba *et al*., 2024). Taken together, these results indicate that despite the well‐documented influence of soil physicochemical parameters (Bahram *et al*., 2018; Delgado‐Baquerizo *et al*., 2018), host genotype (Yadav *et al*., 2023), and their interaction (Berg & Smalla, 2009), inoculated microbiota can effectively alter rhizosphere community structures. Although M and E are not strictly independent – since the microbial inoculants were derived from the same soils defining the abiotic environments – these results about bacterial and fungal rhizosphere communities at the end of the experiment support the relevance of this specific setup for estimating plant trait microbiability.

**Fig. 2:**
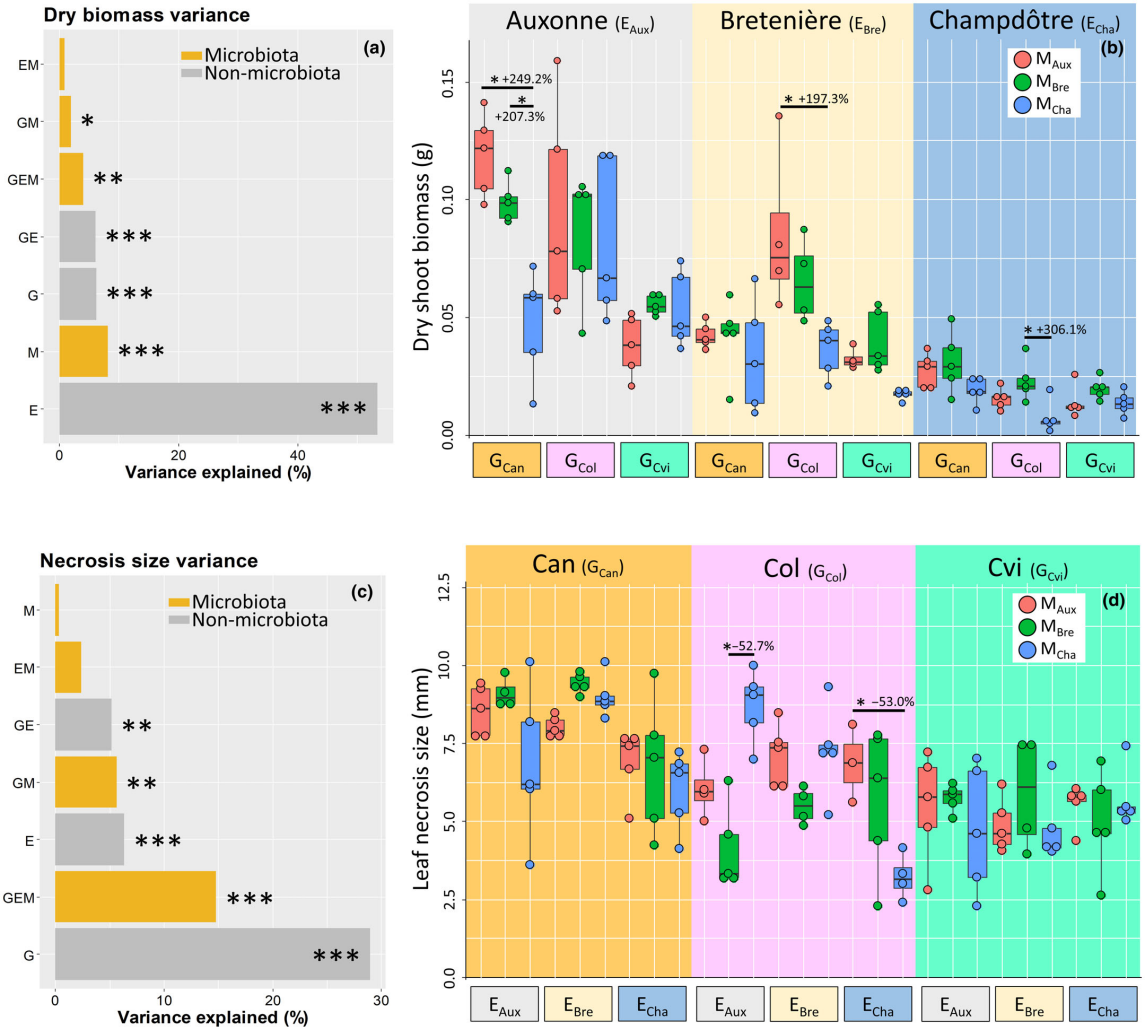
Shoot dry biomass of *Arabidopsis thaliana* (a, b) and necrosis size due to *Botrytis cinerea* inoculation (c, d). Proportion of variance explained by plant genotype (G), soil (E), and microbiota (M) alone or in interaction (a, c). Boxplots of the 27 modalities represented according to the soil for biomass (E = main effect) and according to the genotype for necrosis size (G = main effect). The different colors (red, green, and blue) represent the different microbiota (M_Aux_ for Auxonne, M_Bre_ for Bretenière, M_Cha_ for Champdôtre) inoculated on each genotype and soil. *Arabidopsis thaliana* genotypes: Can, Canary Islands; Col, Columbia; Cvi, Cape Verdi Islands. *n* = 132 for the biomass, and *n* = 128 for the necrosis size. Statistical significance was assessed via Tukey's HSD tests (*P* < 0.05), within each of the three soils for the biomass (b) and within each of the three accessions for the necrosis (d). For clarity sake, only significant changes underscoring an effect of the microbiota (M) were displayed (stars). Boxplot whiskers and horizontal bars are representing the quartiles and median, while dots are representing the data points. Significance code: *, *P* < 0.05; **, *P* < 0.01; ***, *P* < 0.001.

The power simulation showed that our design allows us to detect potential effects of most of the terms of the G × E × M model (Fig. S2), since the simulated number of replicates to reach a power of 80% was below that of our experimental design, except for three interaction terms of the biomass data that needed more replications than what we actually had (G × M, E × M and G × E × M). Nevertheless, we found by chance a significant effect for G × M and G × E × M interactions (Fig. 2). These significant interactions, as well as the corresponding estimates of explained variance, should therefore be interpreted cautiously and require confirmation with a higher level of replication. In future studies of this kind, performing a power analysis would help determine which (interaction) effects can realistically be detected and guide the design of an optimal experimental setup. This includes choosing the appropriate number of genotypes, microbiota, environments, and biological replicates required for robust and reliable detection of effects.

Shoot biomass was well explained by the three factors and their interactions (80.5% of variance explained), mainly shaped by the effect of environment (53.4%, Figs 2a, S3), due to differences in soil properties (Table S2). The following more influential effects were M (8.1%, Figs 2a, S4) and G (6.1%, Fig. 2a). When summing all its significant contributions (M + G × E × M + G × M), M was involved in 14.1% of the variance, and G in 18.2%. The simple M effect was thus higher than previous estimates for shoot and root lengths of *Lotus japonicus* (0.8% and 4%, respectively). But when summed with all significant interaction effects, it was comparable (M + G × E × M + G × M, 7.0% and 17.5% for shoot and root lengths, respectively) (Bamba *et al*., 2024). M_Auxonne_ and M_Bretenière_ had similar effects on biomass, whereas M_Champdôtre_ was associated with significantly lower biomass (M_Auxonne_: +144.6% and M_Bretenière_: +144.7% compared to M_Champdôtre_; Fig. S4).

The dominant influence of E over M and G likely reflects differential soil fertility: the lowest shoot biomass was observed in the soil of Champdôtre, with the lowest N, P, K, and C content, and the highest shoot biomass was observed in the Auxonne soil which had the highest K content (however, the Bretenière soil had the highest N and P content). Other properties like texture, pH, CaCO_3_, and CEC could also interfere. M effect may be due to the involvement of microbiota in nutrient cycling and G effect due to differences in nutrient uptake efficiency.

The G × E × M interaction explained only 4.0% of the variance (Fig. 2b), with notable genotype‐specific responses depending on the soil: in E_Auxonne_, only G_Can_ was affected by M (M_Auxonne_: +249.2%, M_Bretenière_: +207.3% vs M_Champdôtre_); in E_Bretenière_ and E_Champdôtre_, only G_Col_ responded to M (M_Auxonne_: +197.3% and M_Bretenière_: +306.1% vs M_Champdôtre_). Hidden by the G × E × M interaction, there was a small significant G × M interaction (2.0%), confirming that genotypes were not responding in the same way to M across all soils (Fig. S5).

Taken together, these findings suggest that microbiota effects on shoot biomass were of the same importance as G effects, primarily genotype‐independent and mediated through resource availability and soil context.

Pathogen resistance was strongly modulated by host genotype, with microbiota acting through genotype‐specific interactions. In total, 63.5% of the variance in leaf necrosis size was explained by the model. The most influential factor was G (28.9%), followed by G × E × M (14.8%), E (6.3%), and G × M (5.6%, Fig. 2c; Table S1). M had no significant effect alone. However, when summing all its significant interactions, M accounted for 20.4% of necrosis size variance. This is higher than G × M and G × E × M contributions observed for *Lotus japonicus* traits (4.1% and 2.0% for shoot length, and 6.5% and 4.1% for root length, respectively) (Bamba *et al*., 2024). G_Can_ showed the largest necrosis size (+128.0% and +147.6% compared to G_Col_ and G_Cvi_, respectively, Fig. S6), consistently with its higher susceptibility than other genotypes (Denby *et al*., 2004). The strong genotypic influence likely reflects molecular mechanisms of host specificity in plant‐pathogen interactions. Notably, depending on the microbiota applied, G_Col_ can become as resistant as (even more resistant than) G_Cvi_ (Fig. 2d), indicating that M can compensate for a lack of genetic resistance. G_Cvi_ was unaffected by M regardless of E; necrosis size of G_Can_ was mainly influenced by E (e.g. −33.3% effect of M_Champdôtre_ between E_Champdôtre_ and E_Bretenière_); and G_Col_ was the most responsive to M, with reductions ranging from −52% to −53% depending on E (Fig. 2d). The G × M interaction revealed that, with M_Auxonne_, genotypes ranked in the expected order in terms of sensitivity (necrosis size of G_Can_ > G_Col_ > G_Cvi_) (Denby *et al*., 2004), whereas M_Champdôtre_ increased the sensitivity of G_Col_ (G_Can_ ≈ G_Col_ > G_Cvi_), and M_Bretenière_ reduced its sensitivity to the level of the most resistant genotype (G_Can_ > G_Col_ = G_Cvi_) (Fig. S7). Overall, M interfered with pathogen infection through genotype‐ and environment‐dependent pathways, reinforcing the idea that microbiota effects on disease resistance were mediated by the plant host.

Microbiota strongly contribute to plant phenotypic variance and may explain part of the missing heritability in plant traits. We found that microbiability was equal to 8.1% for M alone, up to 14.1% including microbiota‐driven interaction effects for shoot biomass, comparable with G effects. M interaction effects explained up to 20.4% of variance for leaf necrosis size, without a standalone effect of M. This value is close to results in animal systems like dairy cows, in which the gut microbiota explained 13% of methane emission variance, and host genotype 21%; however, both effects being largely independent (Difford *et al*., 2018). Our results highlight the potential of soil microbial communities to contribute to the unexplained phenotypic variance – often referred to as ‘missing heritability’ (Maher, 2008; Danchin *et al*., 2011). Future studies using experimental evolution and transgenerational microbiota transmission (Jacquiod *et al*., 2022) are needed to determine whether this variance is heritable. While host genotype effects on microbiota composition are well established (Yadav *et al*., 2023), our findings call for further research into the heritability of plant traits mediated by microbial communities (Mueller & Linksvayer, 2022).

Despite relatively important M effects observed in our study, experimental choices likely led to an underestimation of microbiability and microbiota‐driven interaction effects. Soils were selected for their contrasting physicochemical properties, notably their contrasted texture (Table S2), and genotypes were chosen based on known differences in pathogen resistance (Denby *et al*., 2004), but microbiota were not selected based on functional properties; instead, they were opportunistically leveraged from selected soils. Their influence might have been greater had the communities originated from soils with renowned effects on plant growth or disease suppression (Weller *et al*., 2002; Sanguin *et al*., 2009). Additionally, inoculating rhizosphere microbiota, rather than bulk soil microbiota, might have increased the effects on plants since rhizosphere soils are typically enriched with plant‐interactive microbes due to host‐driven selection (Lemanceau *et al*., 2017; Rolfe *et al*., 2019). The use of autoclaved (but not axenic) soils could also modulate microbial effects, potentially dampening their expression due to residual endogenous microbiota. In fully sterile systems, inoculants might exert stronger effects but contaminants could modify community structure. In non‐sterile soils, competition may limit the impact of inoculants. Our study represents a proof‐of‐concept in estimating M effects in autoclaved soils. Future studies aiming at assessing M effects in the field would require an interdisciplinary approach integrating agronomy, microbiology and statistical modeling, while mobilizing theoretical frameworks such as the ecology of invasive species in relation to endemic community structure (Liu & Falcão Salles, 2024) and microbiota coalescence (Rillig *et al*., 2015).

Our findings demonstrated that microbiota influenced plant phenotypes differently depending on the targeted plant trait (shoot biomass or pathogen resistance), which has important implications for breeding. Specifically, the trait in question should guide how microbiota are incorporated into artificial selection programs. In standard QTL mapping, breeders typically evaluate 200 genotypes, each with three replicated plots (‘3rep’), across 3 to 6 locations over 1 to 3 yr – equivalent to 3 to 18 ‘site‐year’ (environments, E) (Gutiérrez *et al*., 2015). This results in 1800 (200G × 3E × 3rep) to 10 800 (200G × 18E × 3rep) plots. Introducing M as a third factor can oversize the experimental design. To illustrate this crucial point for breeding, we arbitrarily considered 200 genotypes, even if QTL mapping is not necessarily a goal. We considered 3 to 18 distinct microbiota treatments, comparable in scale to ‘site.year’ – each with three replicates.

Based on our results, shoot biomass was mainly influenced by direct microbial effect (M), largely independent of genotype (G) and environment (E) (very weak G × M effect and non‐significant E × M effect), despite strong differences between environments (E effects). In order to identify the most interesting microbial inoculant, screening 3 to 18 microbiota on a single genotype at a single site with three replicates would require only 9 (3 M × 1G × 1E × 3rep) to 54 plots (18 M × 1G × 1E × 3rep)—making initial screening very accessible and scalable. Conversely, microbial effect on pathogen resistance involves dominant G × E × M and G × M interactions. Accounting for these would require scaling up the previous design, by multiplying it by the number of genotypes (200) and site.year (3 to 18)—ranging from 5400 plots (3 M × 200G × 3E × 3rep) to 194 400 (18 M × 200G × 18E × 3rep)—making full designs almost impractical. However, if only E × M or G × M interactions are significant (but not G × E × M), designs remain demanding but feasible. For E × M alone, testing 3 to 18 microbiota across 3 to 18 environments would require 27 (3 M × 1G × 3E × 3rep) to 972 (18 M × 1G × 18E × 3rep) plots. For G × M alone, 200 genotypes tested with 3 to 18 microbiota would require 1800 (3 M × 200G × 1E × 3rep) to 10 800 (18 M × 200G × 1E × 3rep) plots. These remain within reach for medium‐to‐large breeding efforts. Considering that not all environmental conditions, particularly climatic ones, are conducive to pathogen development, this information could be used to reduce the number of environments explored, thereby decreasing the number of E, E × M and G × E × M modalities.

To conclude, identifying traits with limited G and E dependency is key to deploying scalable microbiota‐assisted selection. For traits involving complex G × E × M interactions, targeted experimental strategies or high‐throughput screening will likely be needed before field implementation.

## Materials and Methods

### Plant

*Arabidopsis thaliana* (Brassicaceae), a model organism with a rapid life cycle, extensively characterized at the molecular level and studied for plant–microbe interactions, was used in this study. Three natural accessions were selected based on their documented susceptibility to the necrotrophic pathogen *Botrytis cinerea* (Denby *et al*., 2004): Can_0 (highly susceptible; mean lesion diameter = 9.5 mm, named ‘G_Can_’), Col_0 (moderately susceptible; 8 mm, named ‘G_Col_’), and Cvi (resistant; 6.5 mm, named ‘G_Cvi_’), with Can for Canary Islands, Col for Columbia, Cvi for Cape Verdi Islands. Seeds were sourced from the Arabidopsis Biological Resource Center at INRAE Versailles‐Grignon, Jean‐Pierre Bourgin Institute.

### Soil

Three distinct soils were collected in the Bourgogne Franche Comté region (France, 13 January 2022): a sandy soil (pH 7.38, 13.6 g kg^−1^ organic C; 69.7% sand, 21.5% silt, 8.8% clay) from Prés‐Velot, Auxonne (47.185860, 5.401536), named ‘E_Aux_’; a clay‐rich soil (pH 7.9, 27.1 g kg^−1^ organic C; 9.5% sand, 51% silt, 39.5% clay) from a grassland at ‘Domaine d’Époisses,’ Bretenière (47.235744, 5.099873), named ‘E_Bre_’; a silty, redox‐degraded Luvisol (pH 5.01, 13.0 g kg^−1^ organic C; 54.3% silt, 34.45% sand, 11.25% clay) from Bois de Boutrand, Champdôtre (47.19974, 5.325278), named ‘E_Cha_’; 1 wk before sowing, all soils dried for 2 wk at 20°C and sieved at 4 mm were autoclaved at 120°C for 40 min, left at room temperature for 24 h to let temperature drop back to 20°C, stored at −20°C for 48 h in order to destroy dormant organisms that could become active after heat stress, left at room temperature for 24 h to let temperature warm back to 20°C, autoclaved a second time using identical parameters and let for 72 h at 20°C. Soil water holding capacity was determined gravimetrically: 33.45 g water per 100 g dry soil (Auxonne), 38.55 g (Bretenière), and 34.55 g (Champdôtre).

### Microbiota

To prepare soil microbial community inoculants (M), 4.5 kg of fresh bulk soil (stored at 4°C after harvest), equivalent to 15 g per microcosm, were suspended in volumes of sterile water equivalent to the field capacity of the corresponding soil. Microbial cells were dislodged from soil particles using a rotary shaker (45 min). Resulting soil slurries were allowed to sediment for 1 h, and the supernatants containing only fine particles in suspension were used as our soil microbial community inoculants, without centrifugation or filtration to keep a maximal diversity. Each pot was watered to field capacity (100%) using the soil inoculants (Auxonne soil: +63 ml; Bretenière soil: +79 ml; Champdôtre soil: +65 ml). Half of the inoculants were applied by sub‐irrigation and the other half by surface application to ensure a homogeneous distribution in pots. Since an equivalent of 15 g of dry soil were used to inoculate 275 g of dry soil for each pot, this corresponds at least to an 18‐fold dilution. The low concentration of nutrients or other substances in the inoculum was thus not likely to produce a significant effect on plant growth. However, to assert that observed effects were due to microbes living in the inoculants, controls consisting of live inoculants, sterilized inoculants, and sterile osmotic water were set, allowing us to detangle effects coming from (1) inoculation procedure (sterile water controls), (2) abiotic components in inoculants (sterile inoculants, two rounds of autoclaving at 120°C for 40 min), and (3) activities of inoculated microbes (live inoculants). We found that sterile inoculants had the same effect as sterile water, whereas living inoculants had a significant positive effect (Fig. S8). Since the pH of the Champdôtre soil was the lowest (5.0 instead of 7.9 for Bretenière and 7.4 for Auxonne), we also verified through a side experiment that the inoculant did not modify the pH of the inoculated soils (Fig. S9). Inoculants had a small (+0.8 log), although significant, effect on the soil pH, as compared with pH differences between soils (+2.5 and +3 log for Auxonne and Bretenière when compared to Champdôtre, respectively).

### Pathogen

*Botrytis cinerea* strain BMM (Zimmerli *et al*., 2001), a generalist necrotrophic fungus, was selected for its relevance to *A. thaliana* pathology (Soltis *et al*., 2020). The strain was cultured for 10 d in the dark at 20°C on half‐strength V8 agar at 30 g l^−1^, buffered with 5 g l^−1^ KH₂PO₄ (pH 6). Conidia were harvested in sterile water, filtered through gauze, and diluted to 5 × 10^4^ spores ml^−1^ in ¼‐strength potato dextrose broth (6 g l^−1^). Infections were performed by depositing 6 μl droplets of the inoculum on the four first fully expanded leaves (from the top) per plant, after 5 wk (clay and sandy soils) or 9 wk (silty soil, due to delayed development). Controls received broth only. To facilitate fungal growth, plants were maintained under high humidity (> 95%), dim light, and 18–25°C in sealed mini‐glasshouses. On the day of infection, pots were adjusted to 90% field capacity.

### Experimental design

A fully factorial G × E × M design was employed (Fig. S1): Genotype (G): three levels (G_Can_, G_Col_, G_Cvi_); soil abiotic environment (E): three levels (E_Aux_, E_Bre_, E_Cha_); Microbiota (M): three levels (M_Aux_, M_Bre_, M_Cha_, extracted from each soil). This resulted in 27 unique GEM combinations, each with 5 biological replicates (135 pots ‘without plant pathogen’, Fig. S1). The entire design was duplicated to include infected plants (135 pots ‘with plant pathogen’, Fig. S1), resulting in 54 treatments and 270 pots. The plants that did not receive the pathogen served as mock‐inoculated controls, via an application on the leaf of a control solution containing only the cultivation media without the pathogen. Additional controls were set to verify potential confounding factors due to our inoculation procedure (see ‘Microbiota’ in the Materials and Methods section).

### Growth conditions

Plants were grown in a glasshouse (20°C : 18°C, day : night, 10 h photoperiod; 60% RH) for 5 wk in the Auxonne and Betenière soils, 9 wk in the Champdôtre soil due to a growth delay due to low pH, which was buffered to 7.55 using KOH in osmotic water (0.1 ml l^−1^). Each pot was filled with 275 g of dry sterilized soil. Before sowing, pots were watered up to 100% of field capacity. Three unsterilized seeds were sown per pot. Post‐germination, only one seedling was kept in each pot in order to have the most homogeneous seedling development within each treatment. All pots were then watered three times a week with the same volume of osmotic water (for each soil), including one weighting to adjust pots individually to 90% of field capacity. Spatial positions were randomized three times a week. Upon detection of Sciaridae adults (*Lycoriella* spp.) on yellow sticky traps (without apparent infection of microcosms), *Steinernema feltiae* (Entonem®, Koppert) was applied following manufacturer instructions, solving the infestation issue within 48 h.

### Plant traits

At the end of the experiment (after 5 or 9 wk, see ‘Growth conditions’ in the Materials and Methods section), shoots were cut and dried 48 h at 50°C in an oven (XXL 01.5, France Étuves) to get shoot dry biomass. Leaf lesion diameter was measured on the four leaves per plant inoculated with *B. cinerea* 72 h post‐infection using digital calipers (Digimatic Absolute AOS, Mitutoyo) and averaged per plant.

### DNA extraction, PCR, and amplicon sequencing

At harvest, rhizosphere soil was collected in 2 ml tubes by gently shaking root systems to retain only tightly adhering soil aggregates and stored at −20°C. DNA was extracted from the 270 rhizosphere soil samples obtained from all replicates using 250 mg of material with the DNeasy PowerSoil‐htp 96 kit (Qiagen). DNA concentrations were quantified via Quant‐iT dsDNA HS Assay (Invitrogen) and diluted to 1 ng μl^−1^ in molecular‐grade water. Bacterial (16S rRNA gene, V3–V4 region) and fungal (ITS1 region) communities were PCR‐amplified using the following barcoded primers (Eurogentec) (Marker‐specific primers in bold): 16S Pro341F: TCG‐TCG‐GCA‐GCG‐TCA‐GAT‐GTG‐TAT‐AAG‐AGA‐CAG‐NNN‐N**CC‐TAC‐GGG‐NBG‐CAS‐CAG;** 16S Pro805R: GTC‐TCG‐TGG‐GCT‐CGG‐AGA‐TGT‐GTA‐TAA‐GAG‐ACA‐GNN‐NN**G‐ACT‐ACN‐VGG‐GTA‐TCT‐AAT‐CC;** ITS1F: TCG‐TCG‐GCA‐GCG‐TCA‐GAT‐GTG‐TAT‐AAG‐AGA‐CAG‐NNN‐N**CT‐TGG‐TCA‐TTT‐AGA‐GGA‐AGT‐AA;** ITS2R: GTC‐TCG‐TGG‐GCT‐CGG‐AGA‐TGT‐GTA‐TAA‐GAG‐ACA‐GNN‐NN**G‐CTG‐CGT‐TCT‐TCA‐TCG‐ATG‐C**. Amplicons were indexed in a second PCR and normalized using the SequalPrep Normalization Plate Kit (Invitrogen) to 1 ng μl^−1^ (*c*. 25 ng total DNA). Libraries were pooled by marker and sequenced (2 × 250 bp) on the Illumina MiSeq platform by GenoScreen (Lille, France). Sample demultiplexing used a custom index list. Of the 270 experimental samples, 264 were selected for sequencing, excluding 6 outlier replicates (low DNA concentrations and too little rhizosphere soil recovery, as the plants were too small). The 16S dataset comprised *c*. 12 million reads (mean: 36000/sample; 88.1% on‐target), and the ITS1 dataset *c*. 3.2 million reads (mean: 6400/sample; 58.04% on‐target).

### Bioinformatics

Sequence processing and analysis of 16S and ITS1 amplicons were conducted using the Galaxy platform hosted by the Migale bioinformatics facility (v.4.1.0), in conjunction with the Find Rapidly OTUs with Galaxy (FROGS) metagenomic pipeline (Escudié *et al*., 2018) (v.3.2). Paired‐end reads were merged using FLASH (Magoč & Salzberg, 2011) v1.2.11, with a mismatch tolerance set at 10%. Operational Taxonomic Units were generated through clustering with the Swarm algorithm (Mahé *et al*., 2015), enabling high‐resolution discrimination of closely related sequences. Quality control steps included screening for PhiX contamination and detection of non‐target host DNA from *Arabidopsis thaliana*. For ITS1 reads, the ITSx tool (Bengtsson‐Palme *et al*., 2013) was applied to improve specificity by filtering out sequences with homology to *A. thaliana* ribosomal regions. Taxonomic assignments were made using two curated reference databases: SILVA 138.1 for 16S sequences and UNITE Eukaryote 9.0 for ITS1 sequences. Despite the marginal presence of archaeal (*c*. 0.01% of the 16S total sequences) and non‐fungal (1.3% of the ITS1 total sequences) matches, we decided to keep all OTUs and called the 16S and ITS1 profiles ‘bacterial’ and ‘fungal’ for simplicity. A preliminary analysis was done on the raw data, followed by a quality check based on visual inspection of ordination plots, a beta‐dispersion outlier test based on centroid distances of samples within each treatment and a removal of samples with too low sequences (< 1000). As a consequence, 251 among 264 16S rRNA and 255 among 264 ITS1 profiles were kept. To estimate sequencing depth, rarefaction curves were generated using the vegan package 2.6‐10 (Dixon, 2003), and samples were randomly resampled at 10 000 and 4000 sequences for 16S rRNA and ITS1, respectively, to normalize the profiles (Fig. S10). Samples below these thresholds were not considered for further analysis. Post‐rarefaction, 245/251 samples were retained for the 16S rRNA profiles, and 161/255 for ITS1 profiles. This filtering ensured between 7–10 and 3–9 biological replicates to estimate each ‘G × E × M’ modality for the 16S and ITS1 profiles, respectively. The resulting contingency tables comprised 1979 bacterial and 165 fungal OTUs. The removal of outliers did not change the significance of detected effects (Fig. S11).

### Statistical analyses

All statistical analyses were performed using R software (v.4.2.0; R Core Team, 2023). The dataset was partitioned into two subsets: (1) mock‐inoculated control plants were analyzed to assess plant dry shoot biomass, and (2) pathogen‐inoculated plants were evaluated for health status via lesion diameter size as a proxy for disease severity. For the biomass dataset, all plants were considered (135/135). In the pathogen dataset, 7/135 replicates were excluded based on failure to establish infection, defined by the minimal lesion diameter size threshold (Stage 1 symptoms, ≤ 2 mm) (Van Wees *et al*., 2013). For biomass, normality was verified by checking the distribution of the residuals of each ANOVA model (Shapiro test) and by visual inspection of the diagnostic plots. Because shoot biomass did not initially meet normality assumptions, a Box‐Cox transformation was applied after identification of the optimal transformation using the ‘bestnormalize*’* package (Peterson, 2021). A second check for normality on Box‐Cox transformed data led to the removal of three outliers based on ANOVA QQplots (132/135) in order to achieve normality. Homoscedasticity was validated thanks to a test of Levene (*P* < 0.05). Graphical representations of biomass data, however, report untransformed values (in grams). Necrosis size data were normally distributed, had homogeneous variance and were thus analyzed without transformation. Three‐way ANOVAs were employed to assess the effects of genotype (G), soil environment (E), and microbiota (M) on both biomass and disease severity, after checking homoscedasticity using a Bartlett's test. The proportion of variance explained by each effect was calculated as the proportion of each factor's sum of squares relative to the total sum of squares, including residual variance (Table S1). Post‐hoc comparisons were done amongst treatments from the same soil for the dry biomass, and amongst the same genotype for the necrosis size, using Tukey's Honestly Significant Difference (HSD) test (*P* < 0.05). For clarity sake, only significant changes underscoring an effect of the microbiota (M) were displayed (Fig. 2). All means comparisons within soil (for shoot biomass) or within genotype (for necrosis size) are shown in Fig. S12. To assess the likelihood of observed statistical effects, a power analysis was conducted using a simulation (*n* = 100, superpower package 0.2.4.1, Lakens & Caldwell (2021), Fig. S2).

Changes in microbial community structure were explored with a beta‐diversity analysis based on the Bray–Curtis dissimilarity index, calculated separately for bacterial and fungal communities. A first PERMANOVA analysis was conducted to test the effect of the pathogen inoculation on the structure of microbial communities, revealing no effects (*P* > 0.05). The final variance partition analysis was hence conducted using the following PERMANOVA model for both the bacterial and fungal datasets: Bray–Curtis ~G × E × M, with 1000 group permutations (Table S1). Models were visualized through a distance‐based redundancy analysis (db‐RDA), using the capscale function (vegan)with 10 000 permutations to assert model validity, followed by 3D projection using the vegan3d package 1.4‐1.

## Competing interests

None declared.

## Author contributions

MB: conceptualization, funding acquisition, project administration, resources, supervision, writing – original draft. OC: investigation, formal analysis, methodology, writing – review & editing. CB: methodology, resources. MG: investigation. OL: methodology, resources, writing – review & editing. SJ: methodology, formal analysis, visualization, data management, writing – review & editing.

## Supporting information


**Fig. S1** Experimental design dedicated to assess the G × E × M model.
**Fig. S2** Power analysis for the dry biomass and necrosis size.
**Fig. S3** Effect of the soil abiotic environment (E) on plant dry shoot biomass.
**Fig. S4** Effect of the soil microbiota (M) on plant dry shoot biomass.
**Fig. S5** Effect of genotype × microbiota interaction on plant dry biomass.
**Fig. S6** Effect of genotype on leaf necrosis size.
**Fig. S7** Effect of genotype × microbiota interaction on the leaf necrosis size.
**Fig. S8** Dry shoot biomass of plants inoculated with sterile water, autoclave‐inactivated and live soil microbial inoculant.
**Fig. S9** A side experiment performed to measure pH of leachates from the soils.
**Fig. S10** Raw rarefaction curves of the bacterial and fungal profiles prior resampling.
**Fig. S11** Community structure of bacteria and fungi based on unfiltered non‐rarefied data.
**Fig. S12** Shoot dry biomass of *Arabidopsis thaliana* and necrosis size due to *Botrytis cinerea* inoculation.
**Table S1** Variance partition for the microbial communities and the plant traits.
**Table S2** Main properties of the three soils.
Please note: Wiley is not responsible for the content or functionality of any Supporting Information supplied by the authors. Any queries (other than missing material) should be directed to the *New Phytologist* Central Office.

## Data Availability

The plant trait data, OTU contingency tables, experimental design files, and the R script have been deposited on the Zenodo repository (https://zenodo.org/record/17963488) and are publicly accessible. The raw sequencing files have been deposited in the Sequence Read Archive repository (SRA, 16S data of the biomass experiment: PRJNA1302175; 16S data of the pathogen experiment: PRJNA1302176; ITS1 data of the biomass experiment: PRJNA1302177; ITS1 data of the pathogen experiment: PRJNA1302178) and are fully accessible.
