# Supplementary material for: Quantifying microbiota impact on plant traits for the guidance of breeding programs
Source: New Phytol. 2026 Mar 26;250(6):3524–33. doi: 10.1111/nph.71108 (PMC13193525; doi:10.1111/nph.71108)
Supplement: Supplementary file 1 — Fig. S1 Experimental design dedicated to assess the G × E × M model. Fig. S2 Power analysis for the dry biomass and necrosis size. Fig. S3 Effect of the soil abiotic environment (E) on plant dry shoot biomass. Fig. S4 Effect of the soil microbiota (M) on plant dry shoot biomass. Fig. S5 Effect of genotype × microbiota interaction on plant dry biomass. Fig. S6 Effect of genotype on leaf necrosis size. Fig. S7 Effect of genotype × microbiota interaction on the leaf necrosis size. Fig. S8 Dry shoot biomass of plants inoculated with sterile water, autoclave‐inactivated and live soil microbial inoculant. Fig. S9 A side experiment performed to measure pH of leachates from the soils. Fig. S10 Raw rarefaction curves of the bacterial and fungal profiles prior resampling. Fig. S11 Community structure of bacteria and fungi based on unfiltered non‐rarefied data. Fig. S12 Shoot dry biomass of Arabidopsis thaliana and necrosis size due to Botrytis cinerea inoculation. Table S1 Variance partition for the microbial communities and the plant traits. Table S2 Main properties of the three soils. Please note: Wiley is not responsible for the content or functionality of any Supporting Information supplied by the authors. Any queries (other than missing material) should be directed to the New Phytologist Central Office. [file NPH-250-3524-s001.docx]

New Phytologist Supporting Information

Article title: Quantifying microbiota impact on plant traits for the guidance of breeding programs

Authors: Manuel Blouin, Olivier Crépin, Cécile Blanchard, Milena Gonzalo, Olivier Lamotte, Samuel Jacquiod

Article acceptance date: 3 March 2026

Supplementary table

Table S1. Variance partition for the microbial communities (PERMANOVA) and the plant traits (ANOVA). df = degree of freedom, SoS = sum of squares, R^2^ = variance explained, F index = F statistics.

| **Dataset** | **Bacteria** | **df** | **SoS** | **R^2^** | **F index** | ***P-value*** | **Signif.** |
| --- | --- | --- | --- | --- | --- | --- | --- |
| Bacterial community (PERMANOVA) | G | 2 | 0.515 | 0.00760 | 3.8183 | 4.00E-4 | *** |
|  | E | 2 | 27.651 | 0.40802 | 205.0534 | 9.99E-5 | *** |
|  | M | 2 | 11.985 | 0.17684 | 88.8731 | 9.99E-5 | *** |
|  | GE | 4 | 0.841 | 0.01241 | 3.1172 | 2.00E-4 | *** |
|  | GM | 4 | 0.693 | 0.01022 | 2.5681 | 3.00E-4 | *** |
|  | EM | 4 | 9.943 | 0.14673 | 36.8687 | 9.99E-5 | *** |
|  | GEM | 8 | 1.443 | 0.02129 | 2.6746 | 9.99E-4 | *** |
|  | Residual | 218 | 14.699 | 0.21689 |  | - | - |
| Fungal community (PERMANOVA) | G | 2 | 0.572 | 0.01112 | 1.9795 | 7.10E-3 | ** |
|  | E | 2 | 8.876 | 0.17244 | 30.6987 | 9.99E-5 | *** |
|  | M | 2 | 13.167 | 0.25580 | 45.5404 | 9.99E-5 | *** |
|  | GE | 4 | 0.847 | 0.01645 | 1.4644 | 0.026 | * |
|  | GM | 4 | 0.899 | 0.01746 | 1.5542 | 0.014 | * |
|  | EM | 4 | 6.060 | 0.11773 | 10.4798 | 9.99E-5 | *** |
|  | GEM | 8 | 1.681 | 0.03266 | 1.4536 | 3.90E-3 | ** |
|  | Residual | 134 | 19.371 | 0.37634 |  |  |  |
| Dry shoot biomass (ANOVA) | G | 2 | 8.04 | 0.061 | 16.521 | 5.78E-7 | *** |
|  | E | 2 | 69.92 | 0.534 | 143.659 | 2.00E-16 | *** |
|  | M | 2 | 10.61 | 0.081 | 21.801 | 1.21E-8 | *** |
|  | GE | 4 | 7.98 | 0.061 | 8.194 | 8.62E-6 | *** |
|  | GM | 4 | 2.56 | 0.02 | 2.625 | 0.0387 | * |
|  | EM | 4 | 1.07 | 0.008 | 1.103 | 0.359 | - |
|  | GEM | 8 | 5.27 | 0.04 | 2.707 | 9.46E-3 | ** |
|  | Residual | 105 | 25.55 | 0.195 | - | - | - |
| Necrosis size (ANOVA) | G | 2 | 144.03 | 28.9 | 40.074 | 1.54E-13 | *** |
|  | E | 2 | 31.56 | 6.3 | 8.782 | 3.05E-4 | *** |
|  | M | 2 | 1.51 | 0.3 | 0.419 | 0.659 | - |
|  | GE | 4 | 25.85 | 5.2 | 3.596 | 8.76E-4 | ** |
|  | GM | 4 | 27.91 | 5.6 | 3.883 | 5.64E-3 | ** |
|  | EM | 4 | 11.71 | 2.4 | 1.629 | 0.173 | - |
|  | GEM | 8 | 73.59 | 14.8 | 5.119 | 2.30E-5 | *** |
|  | Residual | 101 | 181.51 | 36.5 | - | - | - |

Significance codes: *, P < 0.05; **, P < 0.01; ***, P < 0.001.

| Town | Locality | Landuse | Coordinates | pH | % sand | % silt | % clay | organic C (g kg^-1^) | Total N (g kg^-1^) | P (Joret-Herbert, g kg^-1^) | K (g kg^-1^) | CaCO_3_  (g kg^-1^) | CEC (cmol^+^ kg^-1^) |
| --- | --- | --- | --- | --- | --- | --- | --- | --- | --- | --- | --- | --- | --- |
| Bretenière | Domaine d'Epoisses | meadow | 47.235744, 5.099873 | 7.9 | 9.5 | 51.0 | 39.5 | 27.1 | 2.23 | 0.736 | 0.0366 | 38.0 | 23.0 |
| Champdôtre | Bois de Boutrand | forest | 47.19974, 5.325278 | 5.0 | 34.5 | 54.3 | 11.2 | 13.0 | 0.74 | <0.004 | 0.0113 | <1 | 4.83 |
| Auxonne | Pré-Velot | meadow | 47.185860, 5.401536 | 7.4 | 69.7 | 21.5 | 8.8 | 13.6 | 1.60 | 0.257 | 0.0457 | <1 | 6.52 |

Table S2. Main properties of the three soils


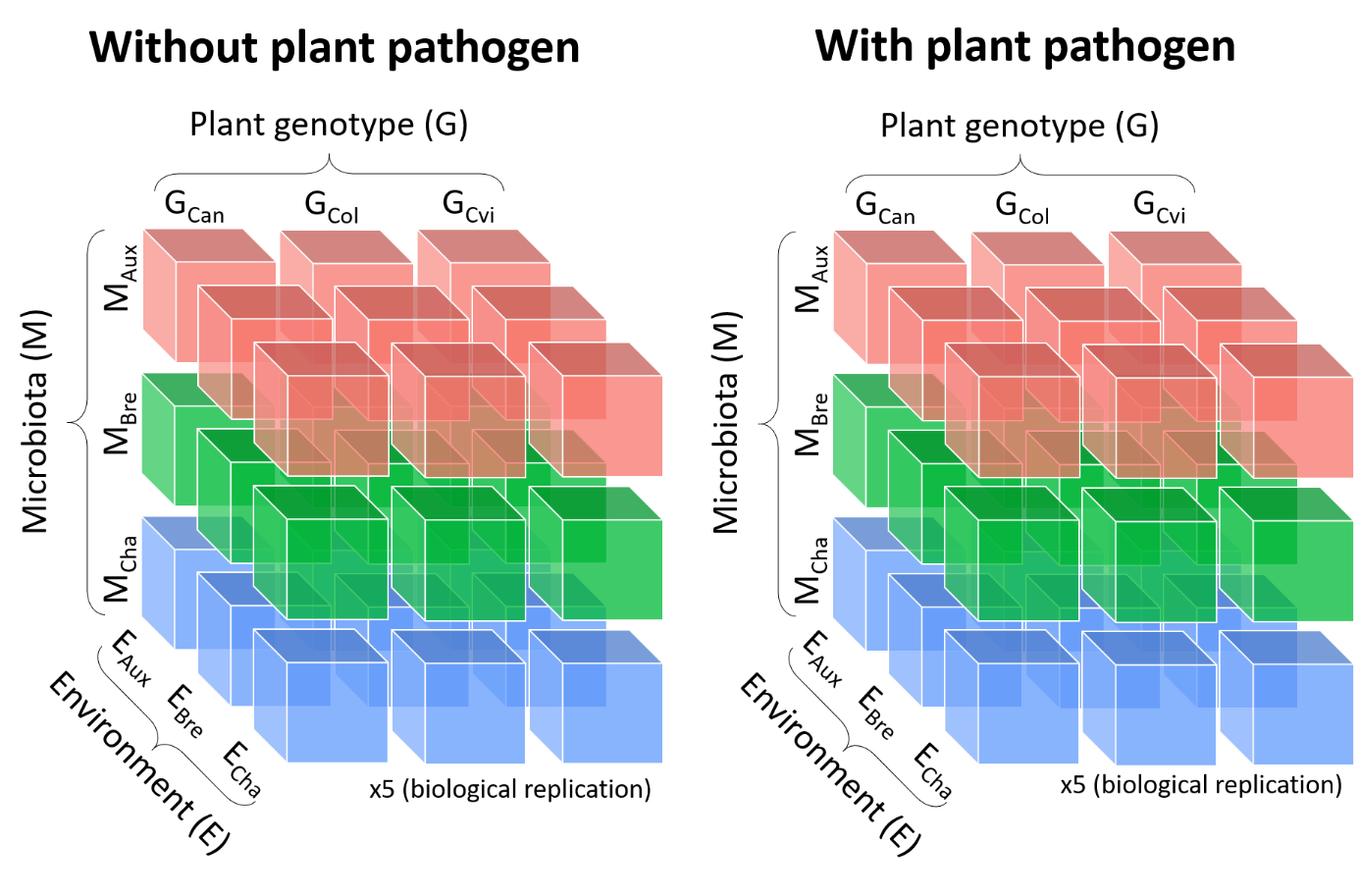


Fig. S1: Experimental design dedicated to assess the G×E×M model. Each cube corresponds to one treatment with five biological replicates. The 3 x 3 x 3 = 27 treatments were replicated twice, in order to measure shoot biomass on plants without pathogen inoculation and necrosis size on another independent plant batch, infected with *Botrytis cinerea*. The different colors (red, green and blue) represent the different microbiota inoculated on each plant genotype and soil. *Arabidopsis thaliana* genotypes: G_Can_, G_Col_, and G_Cvi_ (Can for Canary Islands, Col for Columbia, Cvi for Cape Verdi Islands); soil abiotic environments: Auxonne (E_Aux_), Bretenière (E_Bre_), Champdôtre (E_Cha_); microbiota extracted from the three soils, and re-inoculated in autoclaved soils: Auxonne microbiota (M_Aux_), Bretenière microbiota (M_Bre_), Champdôtre microbiota (M_Cha_).


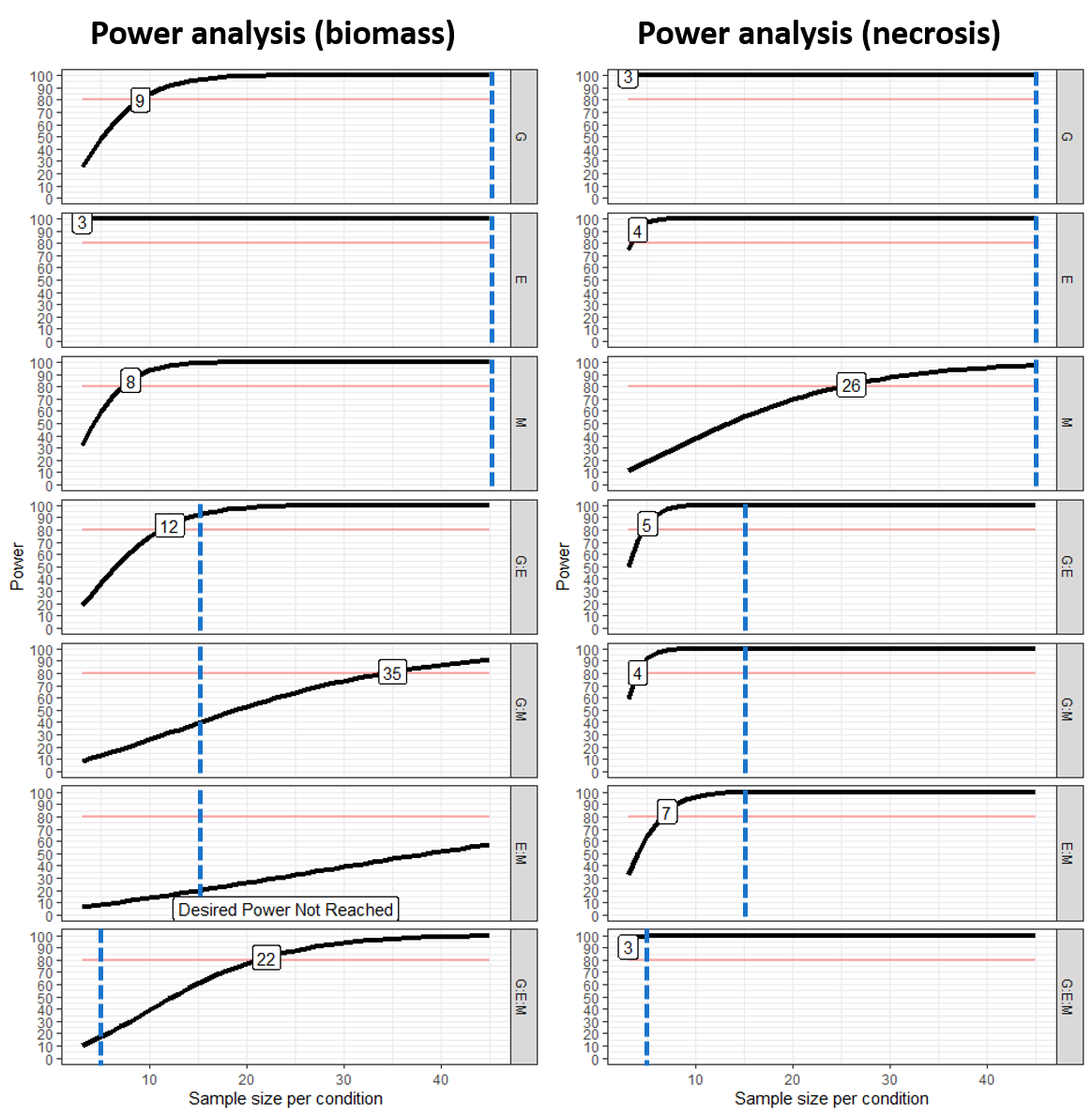


Fig.S2: Simulation of the achieved power based on the number of replicates for the dry biomass and necrosis size data. The figure shows the evolution of power as the number of replicates increases, for all terms in the GEM model. This analysis shows how likely it is to get a significant effect for a given term of the GEM model at a given number of replicates, for the effect sizes and dispersion of data observed in our case. The red line represents the commonly accepted minimal power level (80%). The dotted blue line shows the actual number of replicates used in the experimental design for each terms (n = 45 for ‘G’, ‘E’ and ‘M’ ; n = 15 for ‘GxE’, ‘GxM’, and ‘ExM’ ; n = 5 for ‘GxExM’). Number of simulations: 100.


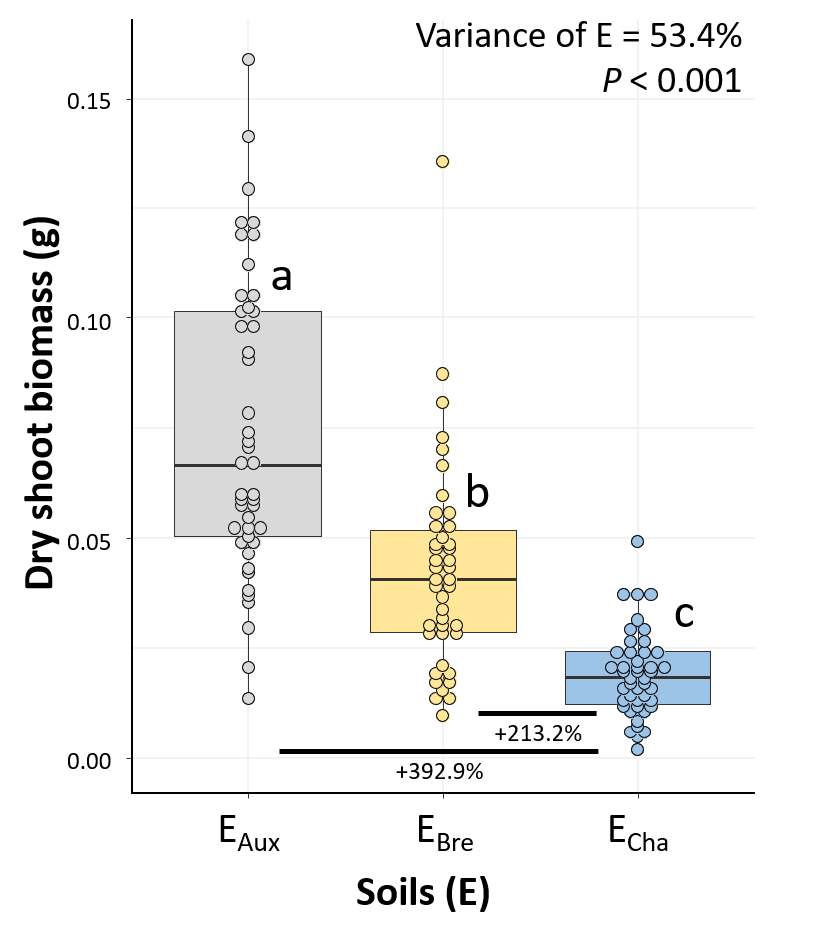


Fig. S3. Effect of the soil abiotic environment (E) on plant dry shoot biomass. The boxplot shows the repartition of the dry shoot biomasses across the three soils (Auxonne: E_Aux_; Bretenière: E_Bre_; Champdôtre: E_Cha_). The amount of variance explained by E and its significance in the full model including all E, M, G and their interactions are provided (n = 132). Different letters indicate significant differences (*p* < 0.05, Tukey‘s HSD). Relative to Champdôtre soil, Breteniere and Auxonne soils had a +213.2% and +392.9% increase in biomass, respectively.


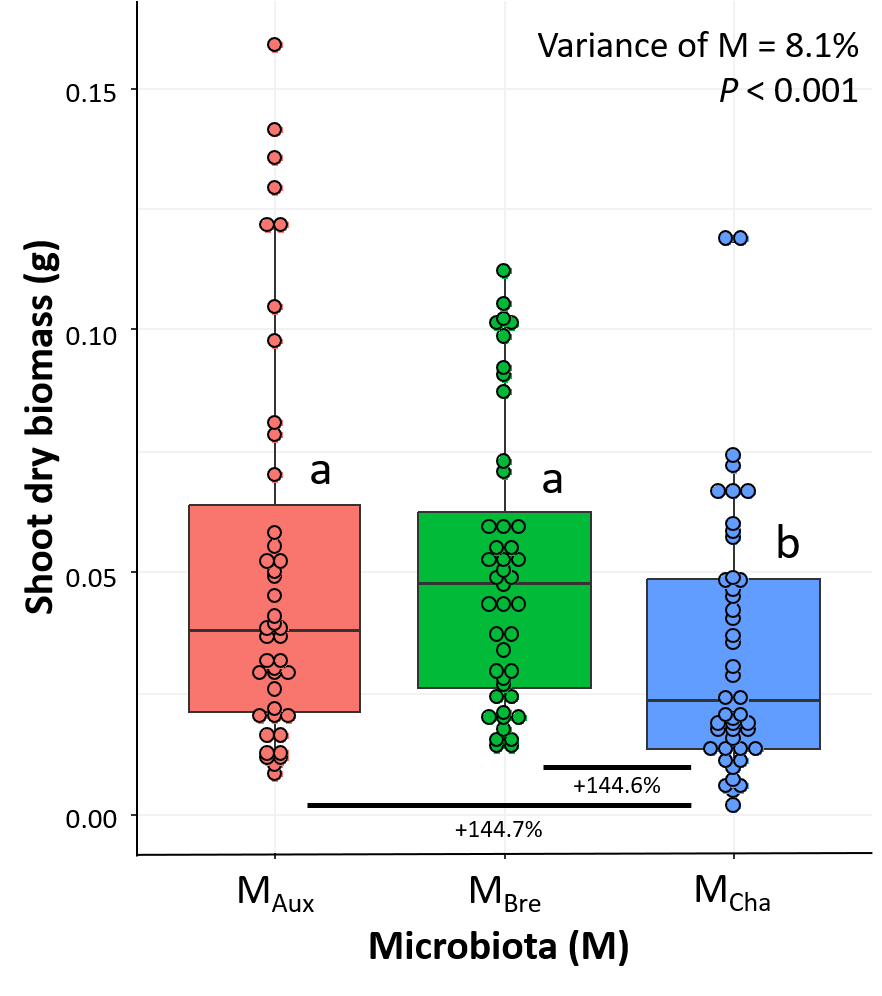


Fig. S4. Effect of the soil microbiota (M) on plant dry shoot biomass. The boxplot shows the repartition of the dry shoot biomasses across the three soils microbiota (Auxonne microbiota: M_Aux_; Brétenière microbiota: M_Bre_; Champdôtre micorbiota: M_Cha_). The amount of variance explained by M and its significance in the full model including all E, M, G and their interactions are provided (n = 132). Different letters indicate significant differences (*p* < 0.05, Tukey‘s HSD). Relative to Champdôtre microbiota, Breteniere and Auxonne microbiota had a +144.6% and +144.7% increase in biomass, respectively.


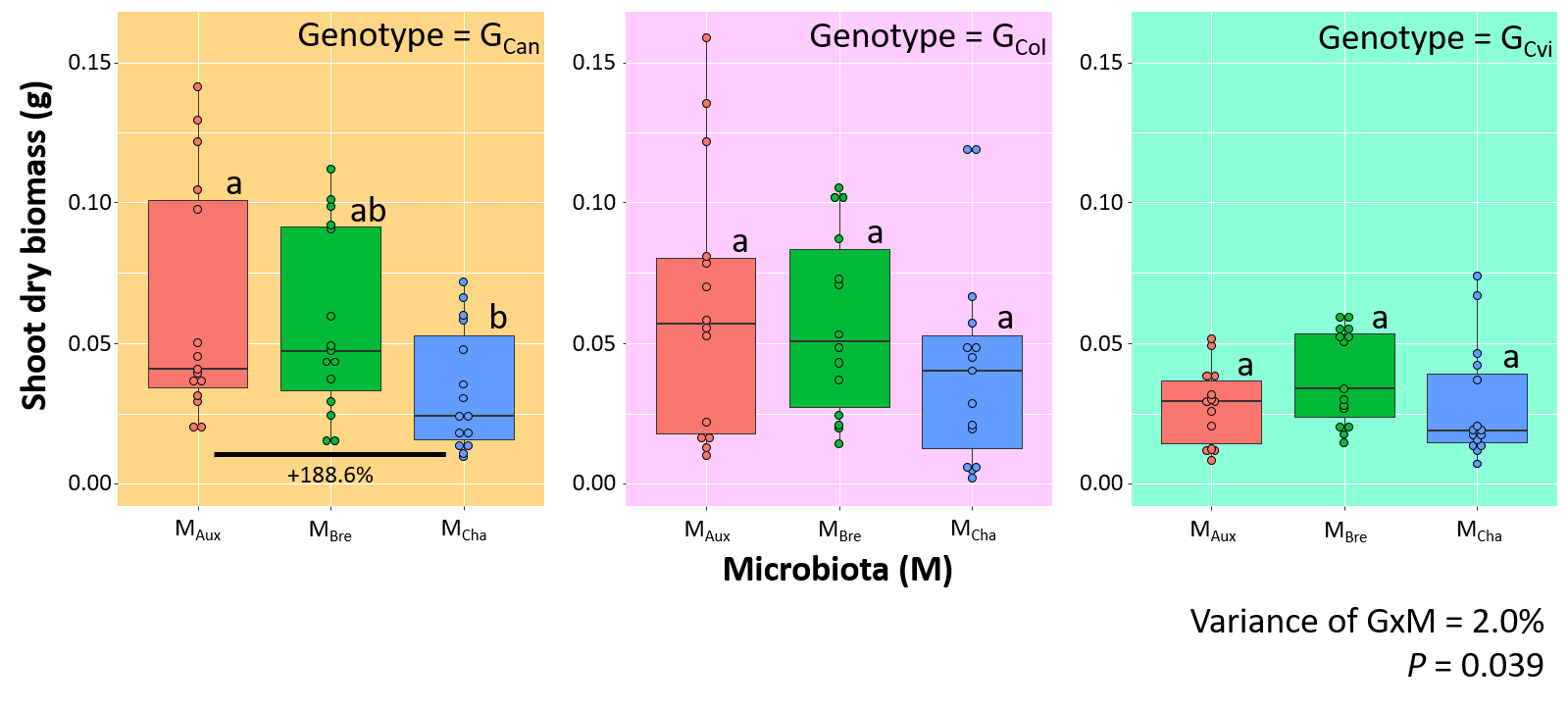
Fig. S5. Effect of genotype x microbiota interaction (GxM) on plant dry biomass. The boxplots show the repartition of the dry shoot biomasses across the three soils microbiota (Auxonne microbiota: M_Aux_; Bretenière microbiota: M_Bre_; Champdôtre micorbiota: M_Cha_) for each plant genotype (Can for Canary Islands, Col for Columbia, Cvi for Cape Verdi Islands). The amount of variance explained by the GxM interaction and its significance in the full model including all E, M, G and their interactions are provided (n = 132). Different letters indicate significant differences (p < 0.05, Tukey‘s HSD). For the Can genotype, and relative to Champdôtre microbiota, Auxonne microbiota had a +188.6% increase in biomass.


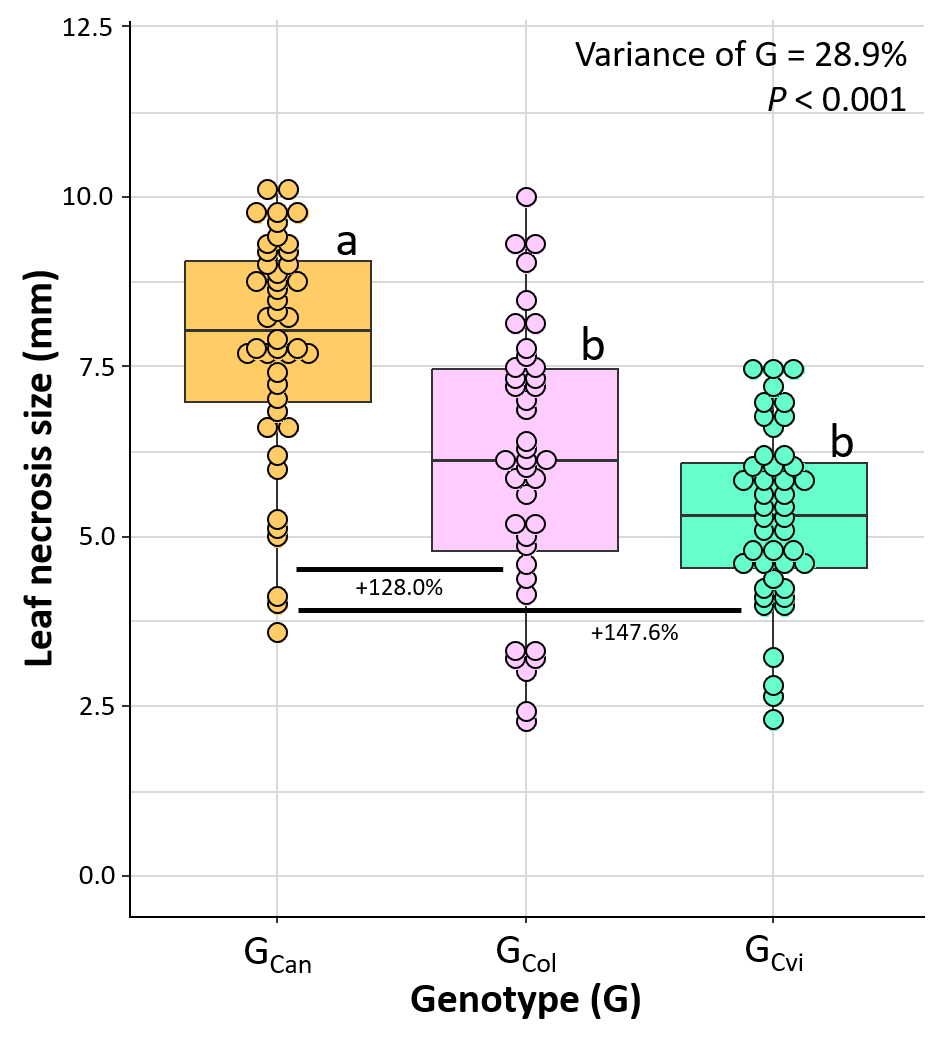


Fig. S6. Effect of genotype on leaf necrosis size. The boxplot shows the repartition of the necrosis sizes across the three plant genotypes (Can for Canary Islands, Col for Columbia, Cvi for Cape Verdi Islands). The amount of variance explained by G and its significance in the full model including all E, M, G and their interactions are provided (n = 128). Different letters indicate significant differences (*p* < 0.05, Tukey‘s HSD). The Can genotype had a +128.0% and a +147.6% necrosis size compared to Col and Cvi.


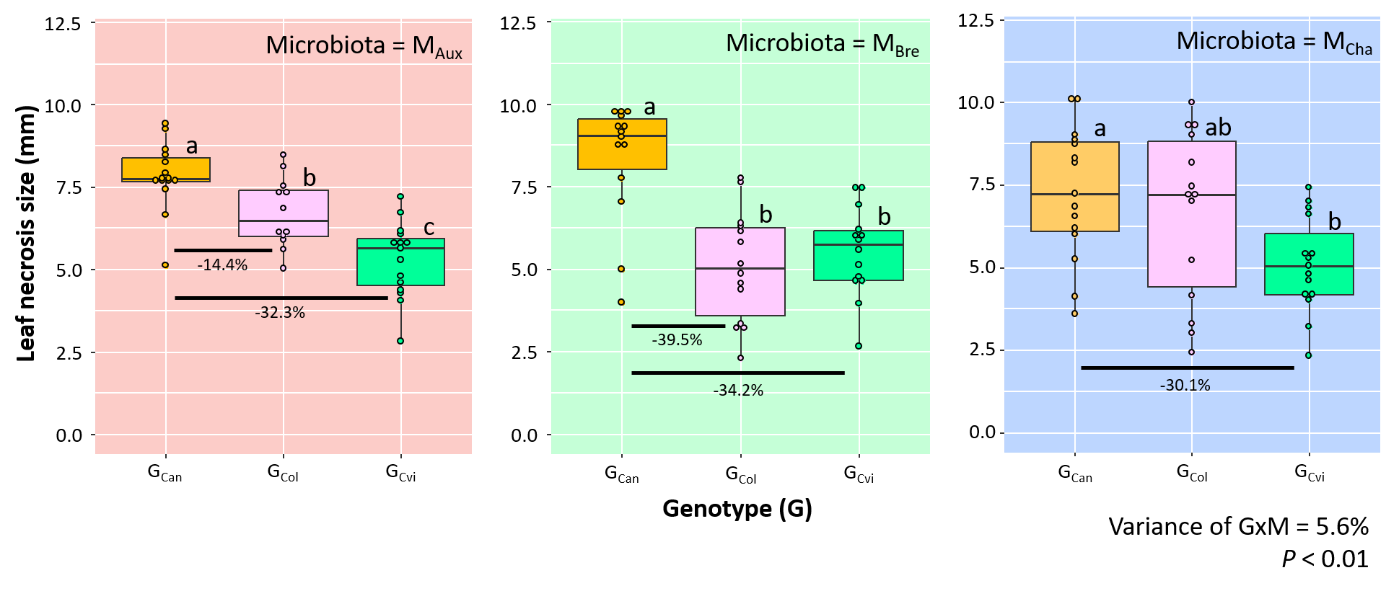


Fig. S7. Effect of genotype x microbiota interaction on the leaf necrosis size. The boxplots show the repartition of the necrosis size across the three plant genotypes (Can genotype: G_Can_; Col genotype: G_Col_; Cvi genotype: G_Cvi_) for each soil microbiota (Auxonne microbiota: M_Aux_; Bretenière microbiota: M_Bre_; Champdôtre micorbiota: M_Cha_). The amount of variance explained by the GxM interaction and its significance in the full model including all E, M, G and their interactions are provided (n = 128). Different letters indicate significant differences (*p* < 0.05, Tukey‘s HSD). Relative to Can, Col and Cvi had, respectively (i) -14.4% and -32.3% reduction in necrosis size with the Auxonne microbiota; (ii) -39.5% and -34.2% reduction in necrosis size with the Breteniere microbiota; (iii) -9.0% and -30.1% reduction in necrosis size with the Champdôtre microbiota.


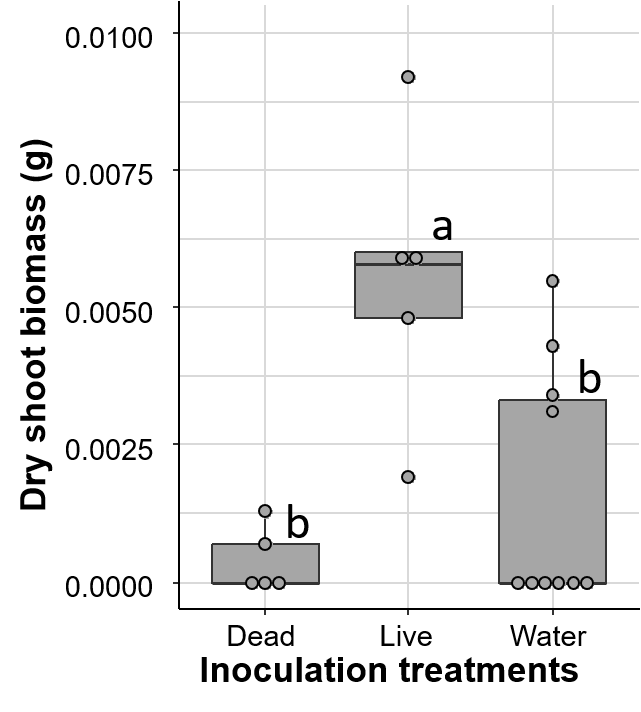


Fig. S8: Dry shoot biomass of plants inoculated with either an autoclave-inactivated soil microbial inoculant (Dead), a live soil microbial inoculant (Live) or sterile water (Water). The experiment was done in the same experimental unit than the main experiment. Since it was not feasible to test the three conditions for all treatments, we did it with the *Arabidopsis thaliana* cv Columbia, in the soil of Champdôtre, with the microbiota originating from the soil of Champdôtre. Statistical differences were inferred with multiple Kruskal-Wallis tests, with a *p*-value adjustment using the False Discovery Rate method (FDR-adj *p* < 0.05). Statistically significant differences are indicated by the different letters. n_Dead_ = 5, n_Live_ = 5, n_Water_ = 10.


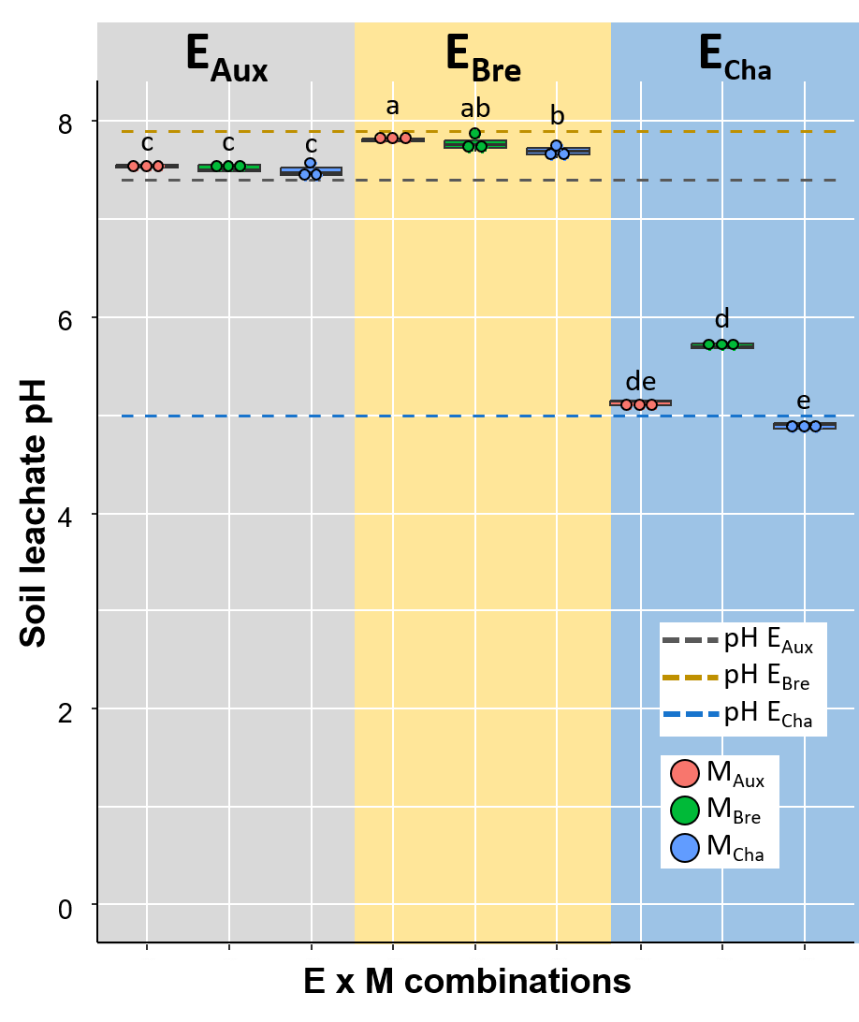


Fig. S9: A side experiment performed to measure pH of leachates from the soils of Auxonne (E_Aux_), Bretenière (E_Bre_) and Champdôtre (E_Cha_) after inoculation with the three inoculants (M_Aux_, M_Bre_, M_Cha_). pH of Champdôtre soil was not buffered with KOH, as in the main experiment. The pH was measured on 15 ml of soil leachate collected in a cup below the pots after watering from the top. Colored dotted lines indicate the pH levels of the three fresh soils. Statistical differences were inferred with multiple Kruskal-Wallis tests, with a *p*-value adjustment using the False Discovery Rate method (FDR-adj *p* < 0.05). Statistically significant differences are indicated by the different letters. n = 3 per treatment.


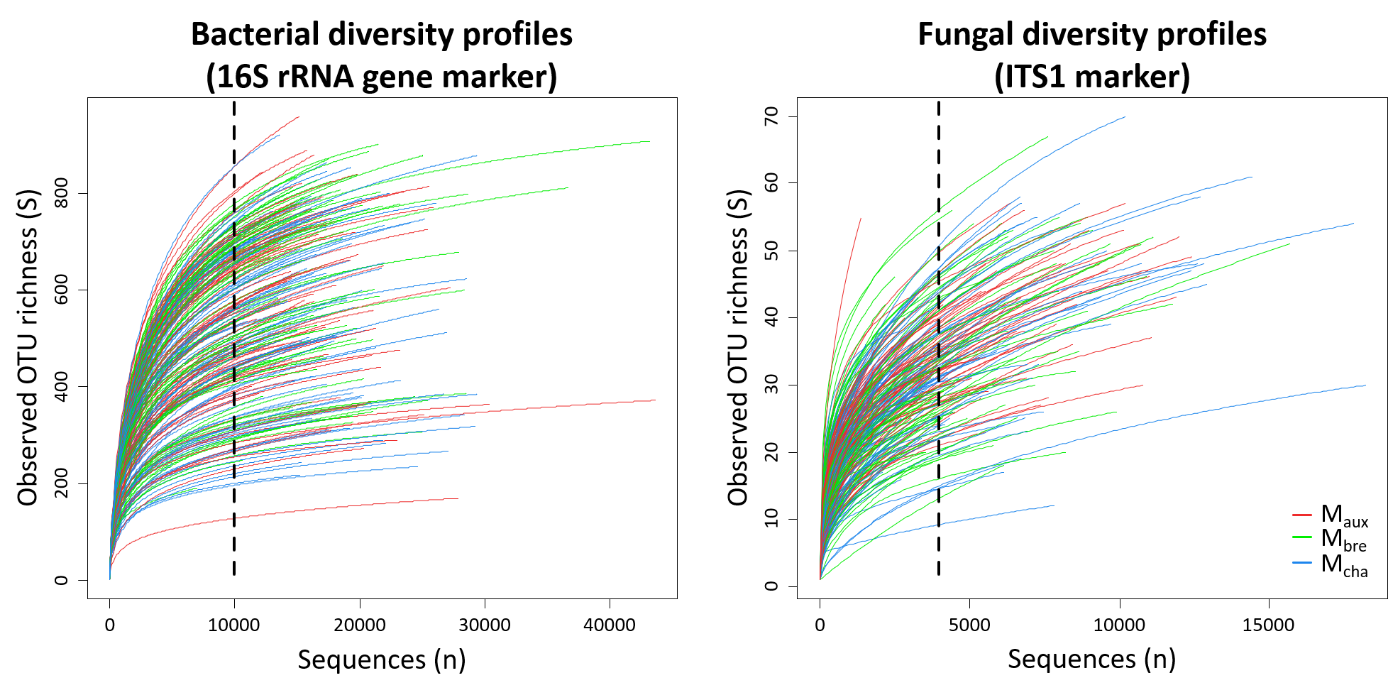


Fig. S10: Raw rarefaction curves of the bacterial and fungal profiles prior resampling. The figure shows the individual diversity detection curves of all samples (OTU richness) as a function of the number of amplicon sequences obtained. The curves are colored based on the microbiota inoculant treatment. The dotted lines indicate the minimum rarefaction level applied to normalize the profiles (10,000 and 4,000 sequences for the bacterial and fungal profiles, respectively). N = 251 (bacteria) and 255 (fungi) samples. M_Aux_: Auxonne microbiota; M_Bre_: Bretenière microbiota; M_Cha_: Champdôtre microbiota)


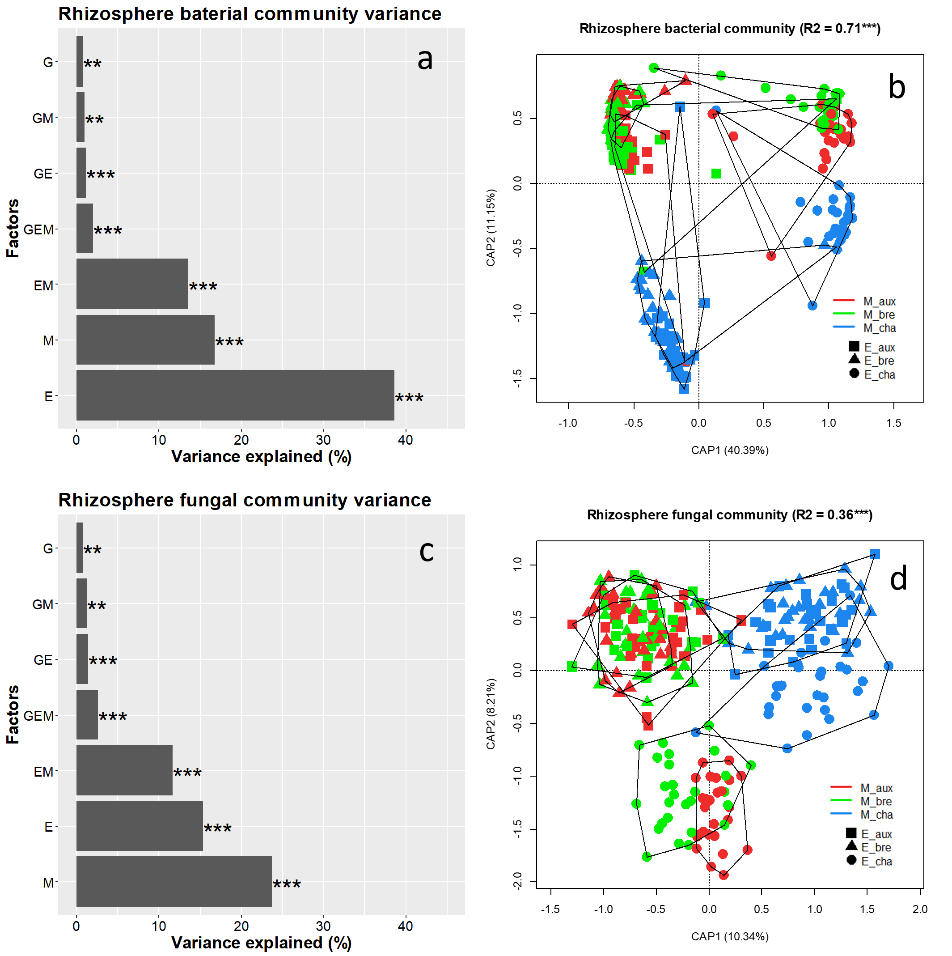


Figure S11: Community structure of bacteria (a, b) and fungi (c, d) based on unfiltered non-rarefied data. Proportion of variance explained by plant genotype (G), soil (E) and inoculated microbiota (M) alone or in interaction (a, c). Distance-based redundancy analysis (db-RDA), based on a constrained model including G, E and M (Bray-Curtis ~ G×E×M, 10,000 permutations) (b, d). The different colors (red, green and blue) represent the different microbiota inoculated on each genotype and soil. The different shapes (squares, triangles and circles) represent the three soils. Microbiota (M): M_Aux_ for Auxonne, M_Bre_ for Bretenière, M_Cha_ for Champdôtre. Due to their limited contribution to the model, and for clarity sake, we did not identify the plant genotypes. N = 261 (16S-rRNA) and N = 257 (ITS1). Significance codes: *, P < 0.05; **, P < 0.01; ***, P < 0.001.


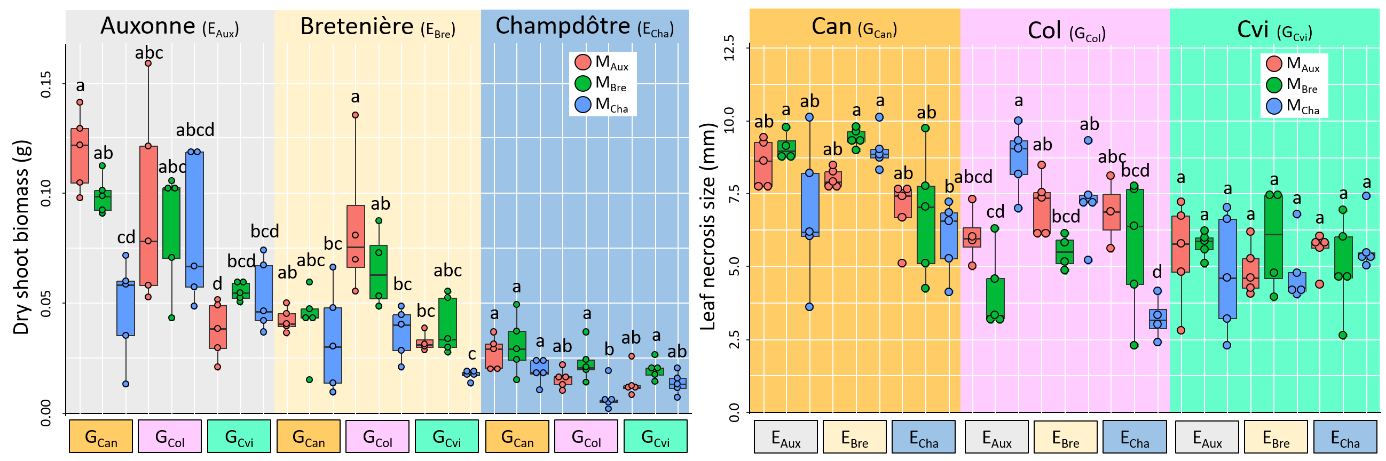


Fig. S12. Shoot dry biomass of *Arabidopsis thaliana* (a, b) and necrosis size due to *Botrytis cinerea* inoculation (c, d). Proportion of variance explained by plant genotype (G), soil (E) and microbiota (M) alone or in interaction (a, c). Boxplots of the 27 modalities represented according to the soil for biomass (E = main effect) and according to the genotype for necrosis size (G = main effect). The different colors (red, green and blue) represent the different microbiota inoculated on each genotype and soil. *Arabidopsis thaliana* genotypes: Can for Canary Islands, Col for Columbia, Cvi for Cape Verdi Islands; microbiota (M) : M_Aux_ for Auxonne, M_Bre_ for Bretenière, M_Cha_ for Champdôtre. N=132 for the biomass, and N = 128 for the necrosis size. Statistical significance was assessed via Tukey’s HSD tests (P < 0.05), within each of the three soils for the biomass (b) and within each of the three accessions for the necrosis (d). Identical letters for different means indicate an absence of significant difference (Tukey HSD test, P < 0.05)
